# Efficiency of remote videoconference-assisted ultrasound education: comparison with standard classroom training

**DOI:** 10.1186/s12909-025-08452-5

**Published:** 2025-12-30

**Authors:** Victoria Vatsvåg, Jo Røislien, Per Kristian Hyldmo, Lars Jacobsen, Nils Petter Oveland

**Affiliations:** 1 Department of Quality and Health Technology, Faculty of Health Sciences, University of Stavanger, Stavanger, Norway; 2 Department for Prehospital Services, Hospital of Southern Norway, Kristiansand, Norway; 3 Department of Anaesthesiology, Stavanger University Hospital, Stavanger, Norway

**Keywords:** Ultrasound, Point-of-care ultrasound, Tele-ultrasound, Remote, Telemedicine, Education

## Abstract

**Background:**

The COVID-19 pandemic necessitated innovative approaches to medical education. This proposed a particular challenge for the teaching of practical skills, such as ultrasound (US) training.

**Methods:**

This study compared the effectiveness of traditional classroom-based US training with that of remote training using teleguided US (Tele-US) among students in the Prehospital Critical Care (PHCC) program at the University of Stavanger, Norway. A total of 44 students were divided into two groups: one received standard classroom training, and the other received remote training because of pandemic restrictions. Both groups underwent a comprehensive one-day US course, including theoretical lectures and hands-on training sessions. Pre-course and post-course tests were used to assess theoretical knowledge, image and video interpretation skills, practical scanning techniques, and clinical decision-making.

**Results:**

There was no significant difference in educational outcomes between the tele-US and classroom groups across all areas of evaluation.

**Conclusions:**

Tele-US appears to be a viable alternative to traditional classroom-based US education, especially in contexts requiring physical distancing or in geographically remote settings.

**Clinical trial number:**

Not applicable.

## Background

 The use of ultrasound (US) as a medical imaging tool has historically been restricted and limited to physicians such as radiologists and cardiologists [1, 2]. The acquisition and interpretation of US examinations are highly user dependent, and one of the main barriers to widespread use is the lack of available equipment, qualified instructors and validated training curricula [3]. In recent years, the development of compact battery-powered US machines that produce high-quality images has facilitated the growth of point-of-care US (i.e., US performed and interpreted by a clinician at the bedside) [4]. This has led to the diffusion of US into a wide range of medical specialties, such as paediatrics, internal medicine, anaesthesiology and emergency medicine, where it is now used by physicians, specialized nurses, general nurses and paramedics. In fact, in the prehospital setting, US is the only readily available diagnostic tool, as pocket-sized hand-held devices make them easy and convenient to use in ambulances or helicopters.

Focused scan protocols have been developed with the aim of assisting clinicians in more rapid and accurate diagnostic decision-making. Point-of-care US (POCUS) images can be obtained immediately and interpreted in relation to the patient´s presenting signs and symptoms. Combining clinical examination with focused goal-directed US scan protocols can confirm and refute life-threatening diagnoses, thereby assisting health care personnel in the initial evaluation and resuscitation of critically ill and injured patients [5]. Two examples are the Focus Assessed Transthoracic Echo (FATE) [6] protocol and extended Focused Assessment with Sonography in Trauma (e- FAST) [7, 8], which are used to identify heart failure, tamponade, pneumothorax and free cavity bleeding in the thorax, abdomen and pelvis, respectively. US is among the most versatile diagnostic tools in modern medicine because it enables clinicians to noninvasively look inside the body for numerous pathologies. Furthermore, it is among the most cost effective and widely available imaging modalities [4].

In line with this development, many universities and teaching institutions are integrating US into their curricula of relevant studies [9–11]. At the University of Stavanger (UiS) in Norway, POC-US is imbedded in the learning objectives of the master’s program entitled “Prehospital Critical Care” (PHCC) since 2014. PHCC students undergo a comprehensive three-day US course in which they are introduced to different POCUS scanning techniques (i.e., hands-on training sessions) and learn how to apply them in various clinical settings.

In 2020 and 2021, the global COVID-19 pandemic halted the continuation of this classroom-based US course because of distancing rules and travel restrictions enforced by The Norwegian Institute of Public Health (NIPH). Consequently, teaching staff at the university had to develop new strategies for delivering both theoretical lectures and practical hands-on training sessions. To teach the use of POCUS at the PHCC master’s program, our solution was to utilize US systems with integrated telecommunication software and interfaces for remote guidance. By connecting our expert instructors and lecturers in their home offices to the students via the internet, we could deliver both theoretical lectures and hands-on training throughout the pandemic. This activity is often referred to as teleguided ultrasound (Tele-US) and allows real-time transfer of the US image, video and audio. In this way, the students can use tele-US during training sessions while receiving instant and continuous feedback on their scanning techniques, image interpretation and diagnostic decision-making skills.

While this teaching approach was successfully implemented, its actual effect is unknown. Few studies have evaluated the effectiveness of Tele-US POCUS training by assessing how well this training compares to traditional—and assumed superior—classroom methods in terms of educational outcomes, skill acquisition and decision-making.

The aim of this study was to evaluate whether remote teaching is noninferior to traditional classroom teaching in the context of ultrasound education, using the forced student restrictions of the pandemic as the basis for an experimental setup.

## Methods

### Study design

This is a noninferiority study assessing whether the educational efficiency of teaching US using tele-US is no worse than that of standard classroom training. Due to logistical constraints, a formal power analysis was not feasible. Instead, we pragmatically aimed to recruit 15–20 students in each group, based on typical class sizes and student availability during the autumn semester.

We set a noninferiority margin of 10%. This means that the collective post-test exam score of the teleguided group should be no more than 10% lower than that of the traditional classroom group. The margin was determined through education judgement, by conferring with a professor of statistics at the University of Stavanger, and aligning with margins used in similar educational studies [12]. The trial setup of the two groups is shown in Table 1.

**Table 1:** Study design

Remote Group	Classroom Group
19 students (PHCC) Recruited autumn semester 2020	25 students (PHCC) Recruited autumn semester 2021 & 2023
Course language: English	Course language: English
Pre-course: 1 day (7 h) e-learning	Pre-course: 1 day (7 h) e-learning
The course-day: Individual pre-course tests supervised by the course leader on Zoom™ (maximum points): a) MCQ (20) b) Picture test (20) c) Video test (20)	The course-day: Individual pre-course tests supervised by the course leader in the classroom (maximum points): a) MCQ (20) b) Picture test (20) c) Video test (20)
Course material & curriculum: 1 day (7 h) remote training. Short individual summary power point lectures on Zoom™ and hands-on training sessions using Butterfly Teleguidance™ on the following subjects/protocols: • FAST-protocol • FATE-protocol • Lung ultrasound • Acute abdomen • Veins/DVT • Veins/IV cannulation	Course material & curriculum: 1 day (7 h) classroom training. Short group-based summary power point lectures and hands-on training sessions on the following subjects/protocols: • FAST-protocol • FATE-protocol • Lung ultrasound • Acute abdomen • Veins/DVT • Veins/IV cannulation
Instructor to student ratio 1:(1-–2)	Instructor to student ratio 1:(5–6)
Equipment: Butterfly iQ™ probe, iPad Pro™, BluePhantom™ IV access trainer, IV needles	Equipment: Butterfly iQ™ probe, iPad Pro™, BluePhantom™ IV trainer, IV needles
Post-course: Individual post-course tests 0–3 days after the course supervised by the examiner on Zoom™ (maximum points) d) MCQ – (20) e) Picture test – (20) f) Video test – (20) g) Practical scanning test – (30) h) US-guided IV insertion test – (10)	Post-course: Individual post-course tests 0–3 days after the course supervised by the course-leader in the classroom for the tests d-f and the examiner on Zoom™ for the tests g-h (maximum points) d) MCQ – (20) e) Picture test – (20) f) Video test – (20) g) Practical scanning test – (30) h) US-guided IV insertion test – (10)

### Participants

The participants were students from the Master’s Program in Prehospital Critical Care (PHCC) at UiS, Norway. This program is open to anyone with a medical degree or bachelor’s degree in medicine, nursing or paramedicine, plus a minimum of two years of full-time clinical prehospital work experience. While it is hosted at UiS in Norway, the program is open to international students and is thus taught in English. The US course was part of the optional 10 credit module point-of-care technology.

In this study, the participants were divided into two groups: those who received training remotely using tele-US, referred to as the *remote group*, and those who received standard classroom training, referred to as the *classroom group*. A convenience sampling approach was used, as the remote group was formed as a direct consequence of COVID-19 pandemic restrictions. Thus, the remote group was intended to receive US training in a classroom setting, but distancing rules and travel regulations prevented the course from going ahead as planned on the university’s premises. The program director for point-of-care technology decided to offer these students remote training rather than cancelling the course, which was the only other available option at the time. Participants in the remote group were recruited during the first pandemic year in the autumn semester of 2020, while the participants in the classroom group were recruited in the two autumn semesters of 2021 and 2023. All the students recruited into the study were novice US users, defined as having no or limited US experience (i.e., previously completed < 25 supervised US scans).

### Faculty

The course leader (VV), a sonographer with 15 + years of experience, was responsible for providing all the summary lectures and supervising during hands-on training in both groups. Efforts were made to keep all teaching sessions as similar as possible, not making any changes to the presentations or tests during the data collection period (i.e., 2020–2023). For the last classroom group in the autumn semester of 2023, two experienced US co-instructors helped supervise during the hands-on sessions to obtain a 1:(5–6) instructor/student ratio. For most of the students in the remote group, teaching was given at a 1:1 ratio, but on two occasions, the ratio was 1:2 for practical reasons.

The course leader (VV) also conducted the pre-course tests in both groups and the theoretical post-course test in the classroom group. The post-course practical scanning tests and US-guided IV insertion tests were all performed after the completion of the courses in both groups by three independent examiners, all with extensive POCUS knowledge. To minimize variability, the examiners used a detailed scoring checklist and jointly evaluated the first two students to calibrate interpretation before proceeding with individual evaluations.

### Equipment

The US machine used during hands-on training in both groups was the Butterfly IQ™ ultrasound probe (Burlington, Massachusetts, United States) connected to a 10.9-inch 4th generation Apple Air iPad™ (Cupertino, California, United States). The remote group received supervision during hands-on training through the TeleGuidance™ tool incorporated in the Butterfly™ app on the iPad. For the duration of the one-day remote course, both the course leader and the students relied on their personal computers connected to high-speed Wi-Fi networks for optimal course implementation. Other equipment used in both groups included an iPad holder, IV cannulas (20G, BD Venflon™ Pro Safety, Helsingborg, Sweden) and an IV training model (Blue Phantom™, 4-branched-vessel training block, CAE Healthcare, Quebec, Canada).

### Course material and curriculum

In both groups the US training was a one-day course with a combination of short summary lectures and hands-on training sessions, covering topics deemed relevant in the prehospital setting. All the students had to complete a seven-hour e-learning program covering all the relevant topics before attending the course day. The e-learning link was sent to the students via e-mail and posted on the UiS student website three weeks prior. As the course leader had no means of checking whether the students had completed the e-learning as instructed, each student had to confirm the completion by signing a declaration form.

### Implementation

**Fig. 1:**
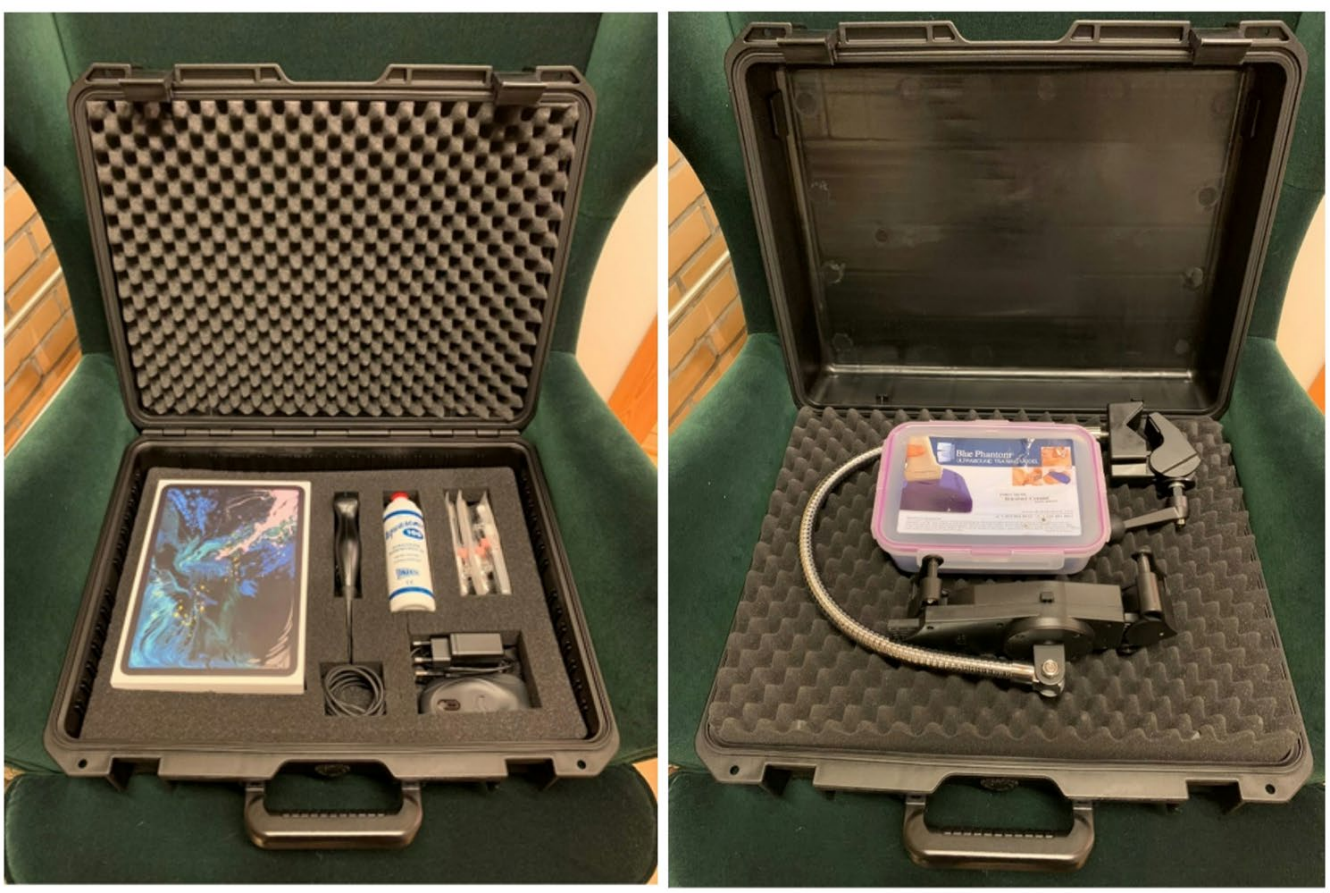
Suitcase with ultrasound equipment sent to all the students in the remote group

For students in the remote group, a suitcase containing all the necessary equipment (Fig. 1) was sent by post to each student prior to the course day. In preparation, each student had to provide a scanning model, i.e., a person, to be available for the duration of the course day. Owing to restrictions related to the COVID-19 pandemic, this person had to have close contact, such as a family member or a close friend. On the course day, the course leader and student connected on both the Zoom™ platform and the Butterfly Teleguidance™ platform and went through the summery lectures and hands-on training sessions, as described in Table 1.

With respect to the students in the classroom group, all the students gathered in a classroom on the university`s premises on the course day and received short summary lectures from the course leader as a single group, followed by hands-on training in smaller groups (i.e., 5–6 students), where they all scanned each other.

All the lectures and equipment were similar for the remote group.

### Pre-course tests

On the course day, the two groups first had to complete individual pre-course tests (Table 2, tests a–c) before beginning the lectures and hands-on training. This was done to establish a reference for measuring the students’ learning post-course completion. The tests were based on topics from e-learning and were divided into three parts: (a) multiple-choice questions (MCQs) (20 questions), (b) picture test (20 pictures) and (c) video test (20 videos). The students were not allowed to use any aides during the tests; they were supervised by the course leader to avoid misconduct and given limited time to answer each question (i.e., 2 min for MCQs, 1 ½ minutes for pictures and 1 min for videos). The maximum score for the pre-course tests was 60 points.

**Table 2:** Summary of test scores for the remote and classroom group

Test	Remote Mean (SD)	Classroom Mean (SD)	Mean Difference (95% CI)	*p*-value*	Cohen’s d
Pre-test (Max score 60)	35.1 (6.1)	36.1 (7.0)	-1.0 (-5.2, 3.2)	0.63	-0.152
Post-test (Max score 60)	44.5 (7.2)	45.1 (7.2)	0.6 (-4.8, 3.6)	0.78	0.083
Total post-test (Max score 100)	73.5 (13.1)	73.6 (13.6)	-0.1 (-8.4, 8.1)	0.97	-0.01

*two-sided independent-samples t-tests

In the remote group, theoretical US questions, US pictures of normal anatomy and pathology and US video clips with pathology were shown as Power Point™ slides in Zoom™, whereas in the classroom group, the same slides were presented via screen display in the classroom. The answers were given directly after the tests to the course leader either on digital answer sheets (remote group) or on paper answer sheets (classroom group).

### Post-course tests

No more than 3 days after the course day, the students took a post-course test (Table 2, tests d–h). Measures were taken to ensure that the students from both groups (i.e., remote and classroom) were evaluated in the same way and in an environment similar to the pre-course tests. All post-course MCQs, pictures and video tests were supervised by one of three independent examiners, using Zoom™ in the case of the remote group and with physical supervision by the course leader in the classroom group. However, all the students from both groups were supervised individually by one examiner, using Zoom™ and Butterfly Teleguidance™ for the practical scanning test and US-guided IV insertion test. Independent examiners were introduced to exclude any favouring biases that might have occurred when the student and course leader who had delivered the lectures and hands-on training knew each other. To further avoid bias, the course leader directly involved in the US training had no access to any pre-course or post-course test results until all the students in both groups had completed their evaluation.

The first three tests in the post-course test—the US questions, US pictures and US videos (Table 2, tests d–f)—were identical to the pre-course tests (Table 2, tests a–c), with an accompanying maximum score of 60 points.

In addition, to further assess the students’ US skills beyond the theoretical components, each student was verbally presented with three separate clinical scenarios: patient [A] with abdominal pain due to aortic aneurism, patient [B] with cardiac arrest due to pulmonary embolism and patient [C] with multiple trauma, including pneumothorax, bleeding in the abdominal cavity and cardiac tamponade. Vital signs (e.g., heart rate, blood pressure, and oxygen saturation) were also provided to the students. The student was then asked to perform the scanning test (Table 2, test g) on a live model (i.e., a person).

The examiners supervised their scanning techniques, including preset selection and gain/depth settings on the Butterfly™ system and probe placement, movements and orientation on the model`s body. Furthermore, if they scanned the model correctly, defined as having the probe in the correct anatomical location with satisfying presets and depth/gain settings, the examiner would show them a US video clip on Zoom™ from that clinical scenario, followed by questions if they could distinguish the pathology from the “real” patient, shown them on their computer screen. The examiners then evaluate their skills for image acquisition, image optimization and diagnostic decision-making and score them following a predefined score card with a minimum score of zero to a maximum of 30 points for all three clinical scenarios [A–C]. Finally, all the students were asked to demonstrate their US-guided IV access skills by inserting an IV cannula into one of the four vessels in a BluePhantom™ training block, first using the in-plane longitudinal technique and then the out-of-plane transverse technique. For this procedural test (Table 2, test h), the students had two attempts per technique, with a pass score of 5 points and a non-pass score of 0 points, depending on whether they could guide the tip of the needle into the centre of the vessel or not. The maximum score for the post-course tests totalled 100 points (Table 1).

The MCQs, picture tests, video tests and clinical scenarios were developed in-house by an associate professor at the university (PhD in ultrasound) based on the e-learning curriculum. All questions were reviewed by faculty with extensive ultrasound experience to ensure relevance and alignment with learning objectives. Question difficulty was controlled through iterative refinement and internal piloting.

### Statistical analysis

Data are summarized as using mean and standard deviation (SD) for normally distributed data and using median interquartile range (IQR) and range for nonsymmetric data. The raw data are graphically illustrated as box plots.

An independent-samples t-test was conducted to compare post-test scores between the remote and classroom groups. Normality was assessed using the Shapiro–Wilk test before applying the test. Effect sizes (Cohen’s d) were calculated for key comparisons to quantify the magnitude of differences beyond p-values. A 95% confidence interval for the difference in mean test scores (Remote vs. Classroom) was calculated to assess noninferiority, with the noninferiority margin prespecified as -10 points.

Individual students’ learning from the pre-course to the post-course tests is shown in a scatterplot. To assess potential learning for each student in the two groups, linear regression models were fitted.

Statistical calculations were performed using SPSS version 29.0.

### Ethical and privacy protection issues

All the students received information about the study and their right to withdraw. Written consent forms were signed. Furthermore, the students were anonymized and assigned a random ID-reference number such that only the course leader could uncouple and link to their real names. The study was approved by the Norwegian Agency for Shared Services in Education and Research [13], and all the data were stored on a secure server at UiS.

## Results

### Group demographics

A total of 19 students were included in the remote group, and 25 students were included in the classroom group. The sex distribution was 9 females vs. 8 females and 10 males vs. 17 males in these two groups.

### Comparison of joint pre- and post-test scores

**Fig. 2:**
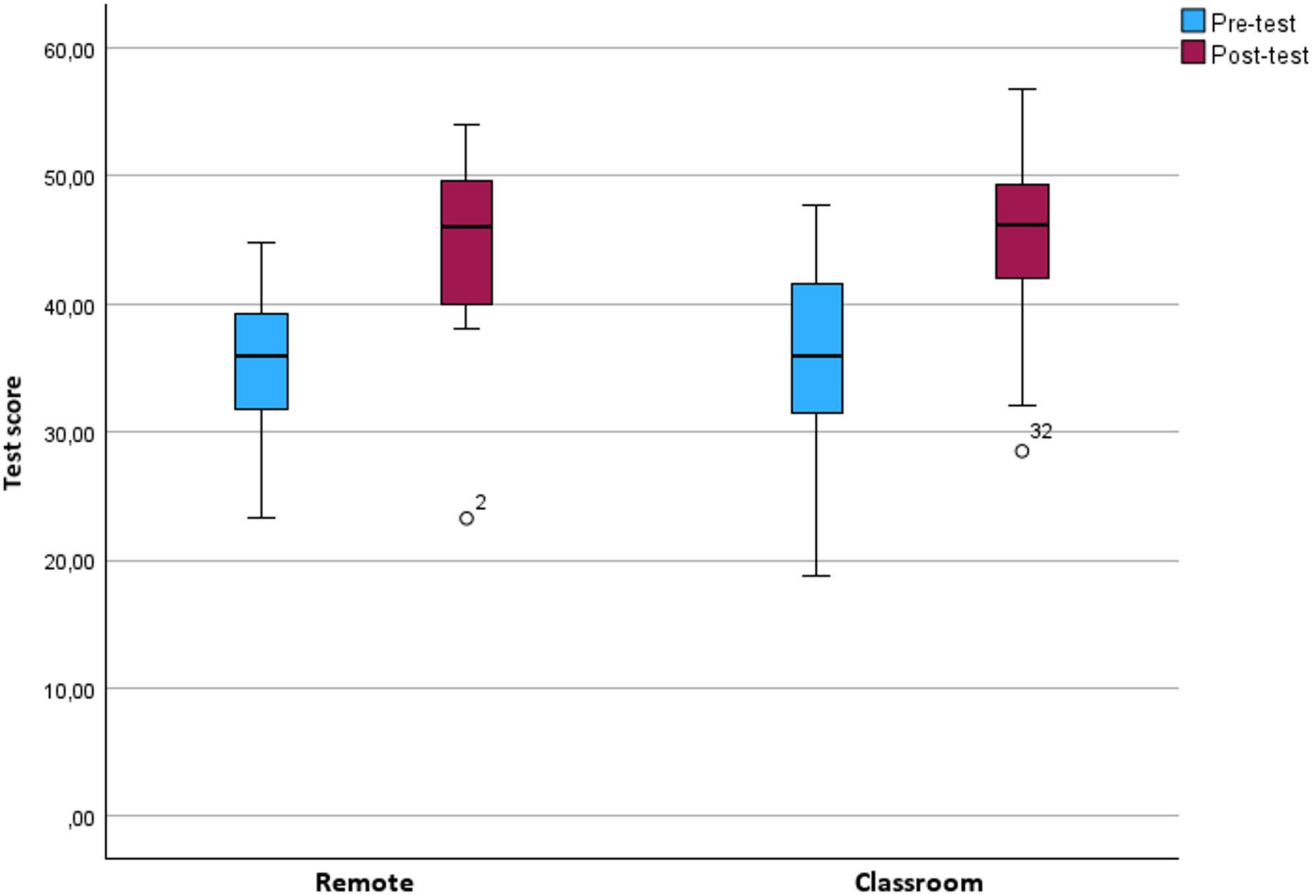
Boxplot of the pre- and post-test scores for the remote and classroom groups. The students were evaluated with identical MCQ questions, US pictures and US videos both before and after the one-day US course

The mean (SD) scores out of a maximum of 60 points for the pre-course tests were 35.1 (6.1) points for the remote group and 36.1 (7.0) points for the classroom group (Fig. 2). This difference was not statistically significant (*p* = 0.63, d= -0,152). Similarly, the mean (SD) theoretical post-course test scores were 44.5 (7.2) points and 45.1 (7.2) points in the remote and classroom groups, respectively (Fig. 2). This difference was not statistically significant (*p* = 0.78, d= -0,083). A complete summary of test scores and statistical comparisons is provided in Table 1.

### Comparison of total post-test scores

**Fig. 3:**
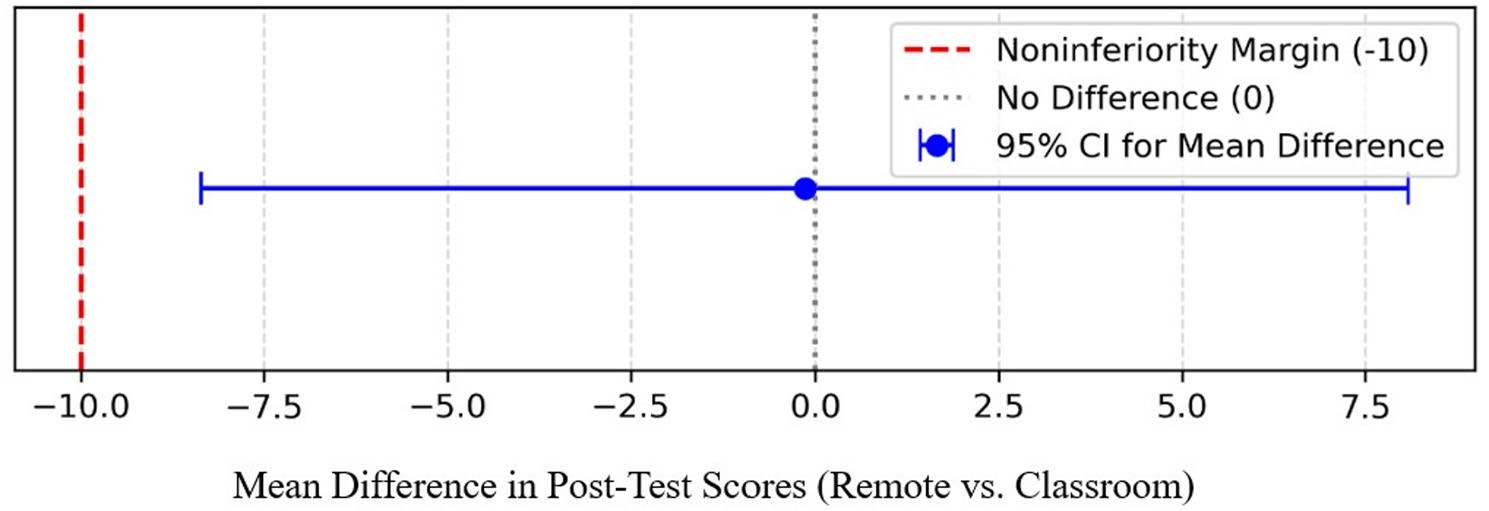
Means and 95% confidence intervals for the difference in means along with the noninferiority margin

The mean (SD) post-test score, which had a maximum of 100 points, was 73.5 (13.1) for the remote group and 73.6 (13.6) for the classroom group (Fig. 3), with a Cohen’s d of -0.01. The mean difference (95% CI) in scores (Remote vs. Classroom) was − 0.1 (− 8.4, 8.1) points (Fig. 3). Given that the lower bound of this CI (-8.4) lies above the noninferiority margin of -10, the criterion for noninferiority was met.

### Learning

**Fig. 4:**
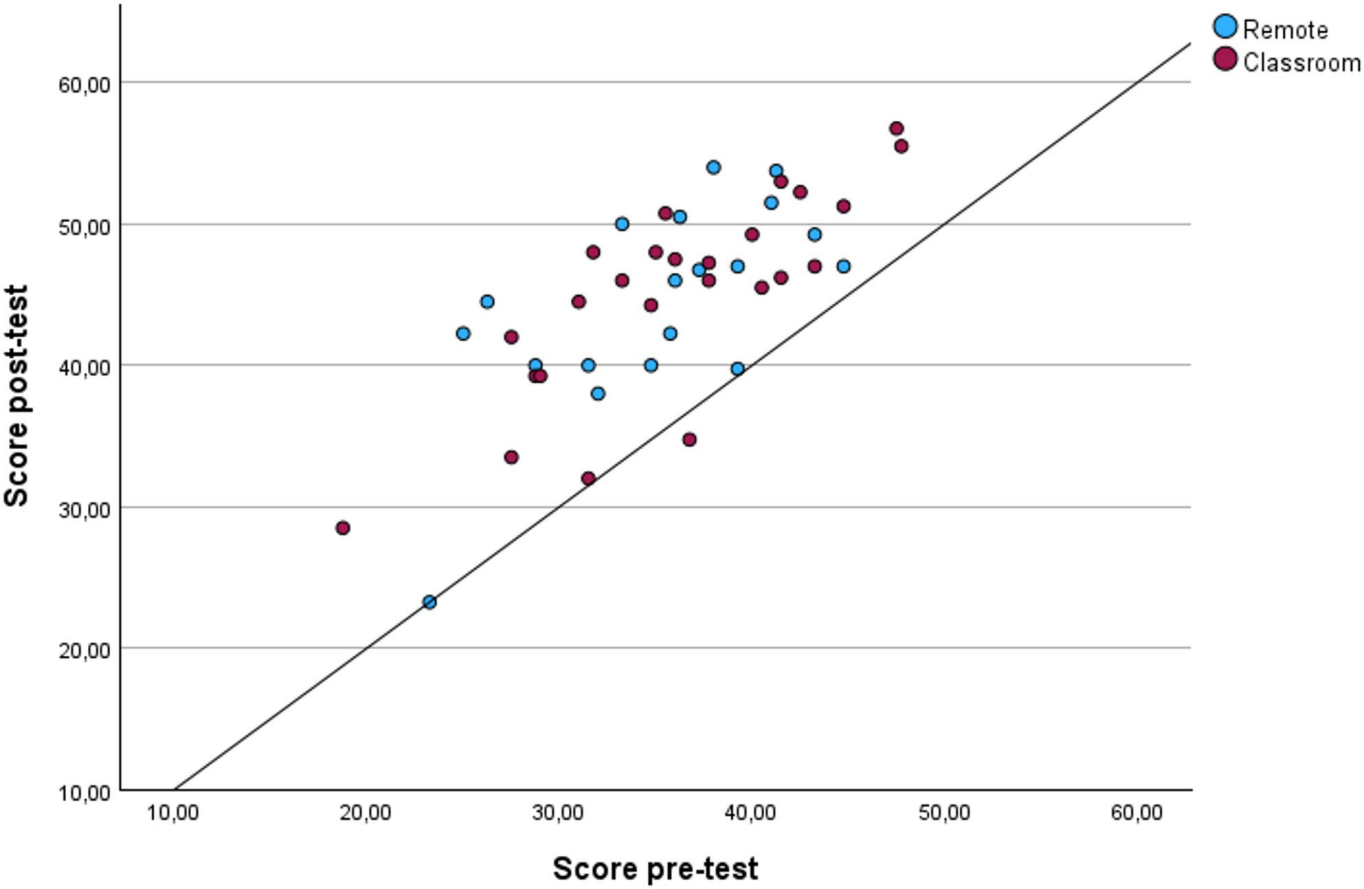
Scatter plot showing the correlation between mean pre- and post-test scores for both groups. Each dot represents one student. The diagonal reference line indicates no change between tests; dots above the line reflect positive learning outcomes, while dots on or below indicate no or negative change

To assess the amount of learning using the two different teaching approaches, we created a scatterplot using each student’s pre-course test score (tests a–c; Table 2) plotted against their post-course test score (tests d–f; Table 2). The results for all the students, colour coded for the remote and classroom groups, are shown in Fig. 4.

Visual inspection of the scatter plot shows that all but a few students in each group (dots on and below the reference line) had a measurable, positive learning effect from the one-day US course, i.e., 17 out of 19 students (89%) in the remote group and 23 out of 25 students (92%).

Fitting a linear regression model to each of the two different teaching approaches, we found slopes (95% CI) of 0.78 (0.33–1.23) and 0.84 (0.57–1.1) for the remote and classroom groups, respectively. The fact that the 95% CIs of the two slopes contain 1 for both groups indicates that learning was equally high for all the students, regardless of their pre-course level.

## Discussion

The findings in this study support our hypothesis that remote teaching is noninferior to traditional classroom teaching in the context of ultrasound (US) education. Based on the prespecified noninferiority margin and observed confidence interval for the difference in post-test scores, remote teaching demonstrated comparable educational effectiveness. Despite the small sample size and corresponding wide confidence interval, the estimated mean difference was well within the prespecified noninferiority margin. Furthermore, a linear regression analysis indicated similar learning for all the students, regardless of the teaching approach used and their pre-course US skill level.

US training programs have traditionally been delivered through standard classroom-based teaching combined with hands-on practice under close supervision [14]. The COVID-19 pandemic required rapid adaption of these teaching methods [15–20].

At the University of Stavanger (UiS), the teaching staff at the point-of-care technology course changed its US training program in the autumn semester of 2020. After careful consideration of distancing rules enforced that year, we decided to move our US course out of the university’s classrooms and into the individual students’ home. Remotely delivering a US curriculum was new to everyone involved, but through careful logistical planning we were able to utilize a new telemedicine tool (i.e., Butterfly IQ™ US probes with TeleGuidance™) and existing communication platforms (i.e., Zoom™) to connect the students and faculty instructors. This innovation became our Tele-US platform, which could be fitted into a suitcase (Fig. 1) and shipped anywhere in the world. The course leader (VV) and support staff had only a few weeks to prepare for all of this, so there was no time or opportunity to test our concept prior to implementation.

The constraints forced upon us by the pandemic did, however, present an opportunity to evaluate the effectiveness of tele-US training compared to traditional classroom US training. The Norwegian Agency for Shared Services in Education and Research approved our study in terms of record time, as it deemed our research important for how to handle future pandemics and remotely deliver education to satellite university locations in Norway.

Telemedicine and POCUS have increasingly merged into a field of tele-US, which has been studied for both diagnostic and educational purposes. Systematic reviews and multiple comparative studies consistently report that remote US training can achieve outcomes comparable traditional classroom instruction [21–34].

Our study builds on this evidence. Comparing our study to the above-mentioned papers, our work is more extensive in terms of curriculum (i.e., included five POCUS protocols for the heart, lungs, abdomen, veins/DVT, e-FAST and one US-guided procedure for IV cannulation). We also used the latest technology in medical communication software (i.e., the TeleGuidance™ tool incorporated in the Butterfly IQ™ US system). As suggested in a case study by Weimersheimer [35], traditional approaches to US education, such as classroom-based instruction or online tutorials, may not be sufficient on their own to provide sustainable, longitudinal training. These multiple cases demonstrate that handheld US systems connected to smartphones and tablets can provide real-time remote bedside scanning supervision through audio and synchronized video and US image transmission. Equivalent, our study shows that with the advent of telemedicine and new US technology, training and the acquisition of new skills can also be supported by a remote faculty of instructors for direct support and image review.

While remote US training requires access to compatible devices and telecommunication tools, the financial and logistical burden may be lower than anticipated. The handheld system used in this study (Butterfly IQ™ with Teleguidance™) is among the most affordable handheld systems on the marked. Additionally, institutions who already own US equipment can adapt existing systems using video capture devices, reducing the need for significant new investments. These factors suggest that scaling tele-US training is feasible for institutions with basic infrastructure and a commitment to integrate remote learning into their curriculum.

Furthermore, compared with previous methods, our method was more comprehensive in terms of the evaluation matrix of the students’ theoretical knowledge, image and video interpretation level, practical US scanning techniques, clinical decision-making competence and US-guided procedural skills. This is, to the best of our knowledge, the first time that students have been exposed to both tele-US and classroom teaching and then evaluated in multiple aspects of US scanning; (I) basic knowledge on how machines and different probes work, scanning modes, presets, gain/depth settings and how to optimize image quality; (II) interpretation of still images and video loops; (III) how to acquire quality US images through good scanning techniques; (IV) the implementation of US into clinical scenarios to diagnose pathology and decide further treatment; and (V) US as a tool to guide clinical procedures. Subanalysis of our data did not reveal any significant difference in the mean score between the remote group and the classroom group when each of these segments was separately assessed.

An important contextual factor in this study was the difference in instructor-to-student ratios between the groups. As previously described, the remote group received highly individualized supervision (1:1 or 1:2), whereas the classroom group had a ratio of 1:5 or 1:6). This discrepancy was due to COVID-19 restrictions during the remote teaching period, which limited the number of students per instructor. Conversely, having the instructor physically present, with the ability to provide hands-on guidance, could be considered an advantage for the classroom group. Both are factors that should be considered when interpreting the findings, and the observed equivalence should not be attributed solely to the teaching modality.

The COVID-19 pandemic made it imperative to rethink new ways of delivering US education and hands-on practical scanning sessions. Previous research suggests that a mixed or blended teaching model is linked to good student learning outcomes [36]. This could include components such as e-learning, online lectures (live or recorded), group discussions, synchronous or asynchronous guidance and feedback on scans and image reviews, use of US simulators and even gamification elements such as quizzes and challenges. In our study, we used elements of this with e-learning (both groups), online live lectures (remote group) or classroom lectures and synchronized guided tele-US with direct feedback on scans and images (remote group) or in-person hands-on training sessions (classroom group). The implementation of remote US training for 19 master’s students in Pre-Hospital Critical Care course in the autumn semester of 2020 gave us experience in terms of the efficiency, benefits, shortcomings and economic impact of tele-US education in comparison to the classroom-based course we had run every year from 2014 to 2019.

Our primary challenge in organizing the course for the remote group was devising a schedule that accommodated the individual availability of each student. As health care professionals during the COVID-19 pandemic, their work schedules were unpredictable and subject to constant change. This posed a significant challenge, especially when trying to schedule teaching within normal working hours. However, remote teaching offers substantial flexibility, as it can be conducted from anywhere at any time with only laptop and internet access. Challenges related to scheduling should, and were, for this reason possible to overcome.

In addition to coordinating the teaching schedule, we encountered some logistical challenges. Equipment had to be tracked to ensure timely delivery to each student. Moreover, the teaching suitcase required restocking and sanitization between each teaching session. This included cleaning all equipment, replacing consumables and ensuring that each component was functional and ready for the next user.

We acknowledge that providing individual students with US equipment in this manner is neither typical nor widely feasible for a remote teaching program. Typically, students have access to ultrasound devices at their educational institutions or clinical workplaces. This is supported by a systematic review by Bui et al. [26], which examined remote US training programs and reported that only 1 out of 24 studies involved settings outside universities or hospitals.

When equipment needs to be shipped, it is advantageous to use compact US devices, such as the handheld probes employed in this project. To facilitate transport and ensure protection, we used hard-case, foam-lined suitcases that were easily handled by postal services.

We initially had some concerns regarding potential internet connectivity issues on the students’ side. Successful delivery of the course depended on each student having access to a stable high-speed internet connection. The Butterfly TeleGuidance™ system used in the course requires either a 4G mobile network or Wi-Fi to function effectively. Fortunately, these concerns proved to be unfounded, as no teaching sessions were disrupted or cancelled because of connectivity issues. This can likely be attributed to Norway’s robust digital infrastructure, with 4G networks covering approximately 99% of the population [37]. However, it is important to note that remote US teaching may pose greater challenges in countries where internet access is less reliable or widespread.

Effectively guiding a learner in the use of an US probe remotely can be challenging, particularly in regard to verbally communicating probe manipulation techniques. For remote US instruction to be successful, it is essential that learners clearly understand verbal directions related to probe movement. These verbal instructions can be enhanced through visual aids. In this project, the US system included a feature that allowed instructors to annotate the real-time US image, a tool that the teaching staff found invaluable for clarifying instructions.

Previous research has proposed strategies for improving verbal communication and instruction for probe movements [38, 39]. However, in our sessions, we did not allocate dedicated time to explicitly teach these movement instructions. In retrospect, we believe that emphasizing this foundational aspect early on could significantly enhance the efficiency and effectiveness of remote US education.

Despite the challenges outlined above, our experience suggests that remote ultrasound teaching can be both time- and cost-effective—particularly for students, who benefit from avoiding travel-related expenses and time away from clinical duties. This reduction in travel also supports sustainability, both by minimizing environmental impact and by making the approach more practical to maintain over time. For instructors, however, one-to-one teaching proved less time efficient. In the future, this limitation could be addressed through strategies such as involving multiple students in the same session, organizing small group learning environments, or incorporating more asynchronous teaching methods to optimize instructor time.

The flexibility of remote instruction—allowing sessions to be conducted from virtually any location and at times that accommodate demanding clinical schedules—has proven to be a significant advantage. With appropriate planning and an emphasis on foundational skills such as probe manipulation, remote US education offers a scalable and accessible alternative to traditional classroom-based instruction, particularly for geographically dispersed or professionally active learners.

### Limitations

We did not perform a formal power calculation for this study. Owing to the constraints imposed by the pandemic, we opted for a pragmatic approach, including one full class of students, which resulted in a relatively small sample size. While this allowed us to initiate a comparison of different teaching methods, the limited number of participants may have reduced the statistical power to detect true differences. Although noninferiority was demonstrated, the precision of the estimates is constrained, and the findings should therefore be interpreted with appropriate caution. Future research on the same topic should aim to conduct a proper power analysis and sample size calculation to strengthen the validity and generalizability of the results.

In addition, the unequal sample size and the use of convenience sampling—both influenced by pandemic-related constraints and the natural distribution of students at our university—represent further limitations. These factors introduce nuances that require careful consideration and highlight the need for additional research to more thoroughly explore the true nature of remote versus classroom-based training.

A factor that may have influenced the findings is the difference in instructor-to-student ratios between the groups, which resulted in the remote group receiving more individualized supervision. We do not know how this difference may have influenced the outcomes. Similarly, the physical presence of an instructor in the classroom setting, allowing hands-on guidance, could also have affected performance. The relative impact of these factors is unclear, and both should be considered when interpreting the results.

As in most educational research, there will always be individual differences between students, such as prior engagement with learning material, motivation and ability to learn. These factors could potentially influence outcomes regardless of group allocation and should be considered when interpreting the findings.

As described in the methods section, the post-test was conducted 1 to 3 days after the course, depending on the availability of both the students and the instructors. Students who completed the test the day after the course may have had an advantage, as the material was likely fresher in their memory than those who took the test after two or three days. However, the distribution of time between the course and the post-test was similar across both groups, minimizing the potential impact of this variation on the overall results.

## Conclusions

Our study supports the hypothesis that teaching US protocols using videoconference-assisted education (tele-US) is not inferior to traditional classroom US training. Tele-US can not only solve the distancing and restrictive challenges of pandemics but also ease the existing problem of a lack of access to both qualified POCUS instructors and diagnostic capabilities in remote locations. Tele-US has the potential to supplement traditional classroom teaching and expand access to US education. However, further longitudinal and multicentre studies are needed to confirm these findings and establish their applicability in different educational settings. 

## Data Availability

The dataset generated and analysed during the study is not publicly available due to institutional data protection policies but is stored securely at the University of Stavanger. Data may be available from the corresponding author on reasonable request and with appropriate ethical approval.

## Abbreviations

FAST: Focused assessment with sonography in trauma
FATE: Focus assessed transthoracic echocardiography
IV: Intravenous
MCQ: Multiple choice questions
NIPH: The norwegian institute of public health
PHCC: Pre-hospital critical care
POCUS: Point of care ultrasound
Tele-US: Teleguided ultrasound
US: Ultrasound
UiS: University of stavanger
